# Railroad Surgeons

**Published:** 1892-04

**Authors:** 


### RAILROAD SURGEONS.

Considerable interest has been manifested in the "railroad surgeon" since the subject was brought up in the American Medical Association, particularly so as they appointed a committee to report on this subject at their next meeting. Their action has caused more or less discussion in the different journals, and will probably cause the subject to be brought before at least some of the State medical associations that affiliate with it.

The tenor of some of the articles, and of a good many of the personal discussions we have heard, has been that the "contract railroad surgeon" should either be censured or expelled from the associations.

As one who has done this class of surgery for several different roads for a number of years under the different methods of compensation, I think my experience entitles me to some voice in the matter, and I think I can speak impartially of the injustice that has been or can be done these surgeons, as I have no connection with any railroad.

In the first place they strike entirely at the "railroad surgeon," when the "accident insurance companies' surgeons" are regulated by a similar tariff. So far as the "contract" part is concerned, every life insurance medical examiner, those surgeons or physicians who have contracts to do work for the various convict camps, cotton mills, machine shops and other places where medical or surgical services are needed, whether under a tariff or a fee bill regulation, *per capita* or monthly, are met by the same objections, and it is a great injustice to particularize between them, and any resolution that might be introduced should apply to all alike.

The conduct of all regular physicians is supposed to be governed by the "*code of medical ethics*" promulgated by the American Medical Association and adopted by all medical associations and societies, whether State or local, and physicians who affiliate with it. Article VII., Paragraph 1 is the only place in the "code of ethics" where there is any word said, or any rule laid down in reference to "pecuniary acknowledgements," and any one who reads it will see that there are absolutely no grounds for the remark that I have so frequently heard, that these men should be expelled, as they are continually violating the "code of ethics" by their contracting to render medical services.

If they have violated no rule nor the code of ethics by which the associations are governed, what right have they to take action of any kind in the matter, whether it be one of censure or expulsion? Of course it may be said that the sentiment of the members are against it. That may or may not be true with the majority of members. Personally, a man has a right to his own opinion in this matter, and to freely express it. But has he any right to cast his vote for a rèsolution, if one should come before an association of which he is a member, that could only result in embarrassing it?

If such a resolution should be passed, it would not only drive many good members from our midst, but might have a further reaching effect.

If a member was expelled, he could by legal action, have himself reinstated as a member. In this connection, I think the older members of the association will recollect having expelled a member on a question of "sentiment" and that they were very glad to reinstate him in order to remove the question of damages done him, by their action, from the courts.

In reference to this question of damages, a member who is even censured, if he could prove damages, could recover a good round amount by legal action against the association. Well, some one will possibly say, if he did, there is no money for him to recover. But he should remember that none or very few medical associations are chartered, and your lawyer will tell you that in case they are not, each individual member is responsible for the amount of any suit brought, and that it can be collected from him.

This is only intended to open up the way to a further discussion of this very important subject, to which there are so many sides to view it from. W. F. W.

